# Study of Temperature on the Corrosion Behavior of Antibacterial Steel by a Large−Scale Multiphase Flow Corrosion Test Loop

**DOI:** 10.3390/ma15217472

**Published:** 2022-10-25

**Authors:** Yunan Zhang, Yongqiang Zhang, Lei Wen, Wei Kong, Yinghua Yang, Jinyang Zhu, Fuhai Liu, Ying Jin

**Affiliations:** 1 National Center for Materials Service Safety, University of Science and Technology Beijing, Beijing 100083, China; 2 Research Institute of Shaanxi Yanchang Petroleum (Group) Co., Ltd., Xi’an 710065, China; 3 Shaanxi Key Laboratory of Carbon Dioxide Sequestration and Enhanced Oil Recovery, Xi’an 710065, China

**Keywords:** large−scale multiphase flow corrosion test loop, antibacterial steel, temperature, CO_2_ corrosion, corrosion couple, electrochemical test

## Abstract

The System #2 flow loop used in this study is a 4−inch−diameter, high−temperature, high−pressure system. In situ corrosion and electrochemical measurements were performed using a homemade flat corrosion specimen and a three−electrode probe. The experiment results show that temperature has an accelerated influence on the corrosion of antibacterial alloy steel. With the increase of temperature and the presence of O_2_ in the environment, a loose and porous corrosion product film was formed on the surface of the resistant steel, which made it easier for the corrosion medium to enter the corrosion product film from the pore, thus accelerating the corrosion.

## 1. Introduction

Although corrosion has been a major cause of failure in oil and gas pipelines, various corrosion control technologies have been developed, such as coatings, inhibitors and cathodic protection. In addition, corrosion resistant alloys have also been developed. Microbial influenced corrosion (MIC) is one of the main types of corrosion encountered in industry, which has attracted extensive attention from researchers at home and abroad [1,2,3]. The use of biocides in the corrosion environment is a common method to control MIC. However, after long−term use of biocides, bacteria will develop drug resistance, and the bactericidal effect will decline. The use of antibacterial steel to control bacterial corrosion has received more attention in recent years. The antibacterial ability of antibacterial steel can be enhanced by adding Cu elements [4,5]. When steel is exposed to a corrosive environments, the copper−rich phase on the surface releases copper ions, which inhibit bacterial growth. Literature shows that the sterilization rate of copper−containing antibacterial stainless steel to some common bacteria (such as Staphylococcus aureus, Escherichia coli, Pseudomonas aeruginosa, etc.) is as high as 90% [4,6]. In order to realize the stable antibacterial function of copper−containing stainless steel, high copper content and proper aging treatments are required. However, the addition and aging of Cu can lead to intergranular corrosion and pitting corrosion of stainless steel, which severely limits its application potential in many fields. The current research suggests that the occurrence of the MIC process is affected by the formation of bacterial biofilm [7,8], and the Cu−containing antibacterial steel has good antibacterial function, which can effectively inhibit the formation of bacterial film in the microbial environment. Additionally, there are fewer reports on the corrosion resistance and corrosion laws of antibacterial steel in oil and gas gathering pipelines.

The research on antibiotics usually conducted in a high−temperature and high−pressure autoclave, which can simulate the high temperature and high−pressure working conditions in the field to a certain extent, but it is difficult to restore the actual flow pattern and velocity in the field. In addition, the current research on antibacterial steel mainly focuses on its ability to inhibit bacterial corrosion, and there is less research on its corrosion law in conventional environments. These reasons restrict the popularization and application of antibacterial steel to some extent.

In view of the above problems, this paper takes the actual working conditions of an oil field in China as the research background, and uses the multiphase flow corrosion simulation loop test device built by USTB (University of Science and Technology Beijing) to carry out the corrosion resistance research of antibacterial steel in a laminar flow environment. Sulfate−reducing bacteria (SRB) are abundant in the formation water of the field, and the SRB content in some blocks is as high as 106 bacteria/mL. The bacteria in the formation water cannot be eliminated with fungicides and can only be prevented by treatment on the surface collection line. Anti−bacterial steel is expected to alleviate and prevent corrosion of the oil field surface gathering pipeline. At the same time, large−scale CO_2_ storage and enhanced oil recovery tests are being carried out in the oilfield. The produced fluid contains a large amount of CO_2_, and the temperature of the collecting pipeline fluctuates widely. It is necessary to further evaluate the CO_2_ corrosion resistance of antibacterial steel at different temperatures, so as to lay a foundation for subsequent field tests. 

Multiphase flow corrosion is one of the great challenges in the oil and gas industry [9,10,11,12,13,14]. A large number of basic and applied researches have been carried out on the multiphase flow corrosion of pipelines in oil and gas fields. On the basis of a full investigation and comparison of multiphase flow corrosion simulation loop systems at home and abroad [15,16,17,18,19,20,21,22], the relevant team from USTB designed and built a multiphase flow corrosion simulation loop test device with large size, multi−function, and flexible combinations, which can evaluate the service safety of large size pipelines in complex multiphase flow environments. 

Thus, this paper takes antibacterial steel as the research object to study the effect of temperature and flow rate on its CO_2_ corrosion resistance in multiphase flow environments. It is expected that this paper will be able to provide support for the application of antibacterial steel in oil and gas fields.

## 2. Experiment

### Material and Solution

The test material is an antibacterial alloy steel; its chemical compositions (wt%) are listed in Table 1. The specimens were ground to 800 grit and then cleaned with deionized water and alcohol. The corrosion solution is a simulated field produced fluid, which is prepared with deionized water and analytical pure chemical reagents. The solution composition of the flow loop test is shown in Table 2. Both the solution and flow loop were deaerated by pure CO_2_ for 24 h. 

## 3. Flow Loop Test

**Figure 1:**
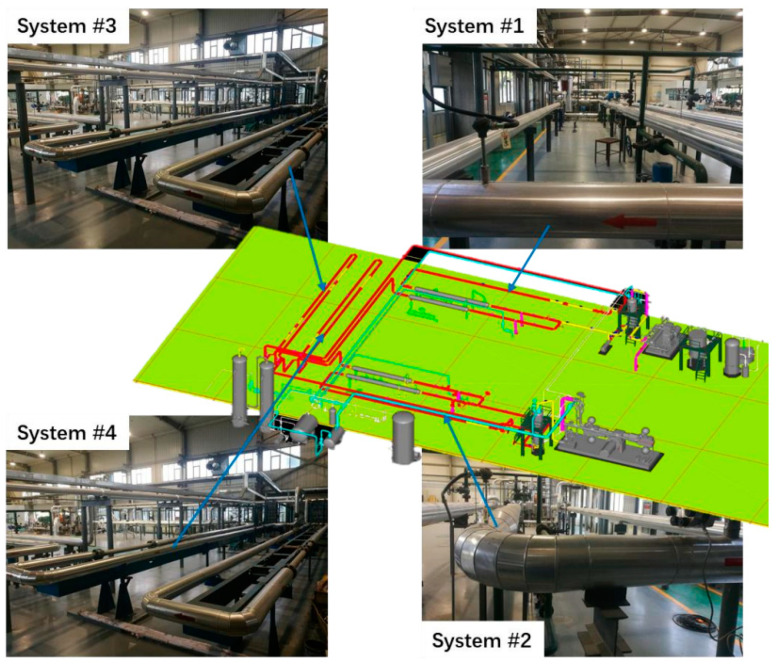
Schematic of the multiphase flow corrosion simulation loop test device.

The test facility consists of four operational large scale multiphase flow loops that are specifically designed for multiphase investigation under realistic temperature, pressure, water chemistry, and flow conditions found in the field. The two high pressure loops are mainly made of Hastelloy C276 (piping, tank, etc.), while the other two low pressure loops are made completely of 316 L stainless steel. They are all approximately 20 m long, 4 in internal diameter, high−pressure, high−temperature, multiphase flow rigs. These flow loops can be used for the corrosion study (sour corrosion, localized corrosion, and inhibitor evaluation). The test section is a 3 m (System #1 and 3) or 2 m (System #2 and 4) long pipe, as shown in Figure 1. The device can realize the dynamic simulation of any two−phase or three−phase environment of oil/gas/water. The highest temperature inside the loop is 140 °C, the highest pressure is 10 MPa, the highest flow rate of gas phase is 17 m/s, and the highest flow rate of liquid phase is 4 m/s. The loop test section can be adjusted arbitrarily from horizontal (0 degrees) to vertical (90 degrees), and has an up−and−down bidirectional simulation system to simulate the actual multiphase flow environment inside the horizontal/inclined/vertical pipeline.

**Figure 2:**
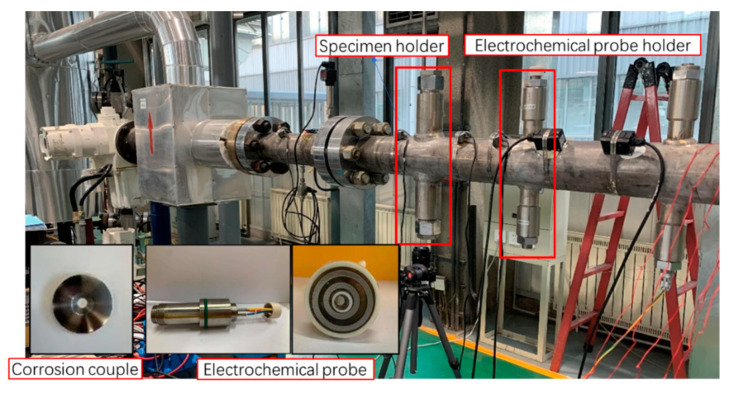
Physical drawing of the test section in the System#2 flow loop.

The System #2 flow loop used in this study, and the device diagram is shown in Figure 1. The System #2 flow loop is a 4−inch−diameter, high−temperature, high−pressure system. Weight loss probes (see Figure 2) are the primary method for corrosion rate measurement because of their ability to perform corrosion film analysis. The coupon was a ring with inner and outer diameters of 6.25 and 30 mm, respectively, and a thickness of 3 mm. The liquid medium of the flow loop is pumped into the medium separator after being pre−pared in the medium distribution tank, and the centrifugal liquid circulating pump is started. The single phase liquid medium operates in a closed loop formed by the pipeline and the medium separator, and reaches the test temperature under the action of the jacket heating controller. At the same time, the high−pressure gas compressor starts to pump the gas into the liquid flow under closed cycle operation, so as to form a gas−liquid two−phase flow. After passing through the test section, it enters the medium separator for gas−liquid separation, and then enters the next closed cycle. The variable frequency regulator of the pump is used to control the liquid and gas flow to reach the specified gas−liquid flow rate. The parameters of the flow loop tests are shown in Table 3.

All electrochemical measurements were performed in a flow loop by using a conventional three−electrode system (Gamry Reference 600+ electrochemical workstation; Gamry Instruments, Warminster, PA, USA). In order to obtain a three−electrode configuration, the central Ag/AgCl electrode column remained unconnected to the other electrodes and served as a reference electrode (RE), with one block of test steel ring acting as the working electrode (WE), and a block of 2205 stainless steel ring as the counter electrode (CE). The three−electrode surface area was 4.15 cm^2^. Electrochemical impedance data was measured at 24 h intervals. For electrochemical impedance spectroscopy (EIS) measurement, the applied potential was Ecorr ± 10 mV, and the frequency range was 100 kHz~10 MHz

A coupon sample and an electrochemical sample clamping groove are set at the bottom of the loop pipeline. The corrosion sample is fixed by a clamp and placed in the clamping groove at the bottom of the pipeline. The working surface of the sample is flush with the inner wall at the bottom of the pipeline. The outside of the sample clamp is sealed with a valve to simulate the real pipeline environment in the wet natural gas pipeline. After installing the sample on the sample slot, high−purity N_2_ was injected for more than 4 h to remove O_2_ in the pipeline and water tank, and then high−purity CO_2_ gas was injected, the pressure was adjusted until the gas pressure reached 5 MPa, and the test temperature was set at 20 °C, 50 °C, and 60 °C for experiments 1, 2 and 3, respectively.

The weight loss method was utilized to measure the corrosion rate. Scanning electron microscopy (MERLIN COMPACT FESEM, Zeiss, Oberkochen, Germany) coupled with an energy dispersive X−ray spectroscopy (EDS) were used to obtain morphology information and the corresponding elemental information. The corrosion morphology of the specimen surface was measured using an optical profiler (Bruker ContourGT−K0; Bruker, Billerica, MA, USA). 

## 4. Results

### Corrosion Weight Loss Analysis

**Figure 3:**
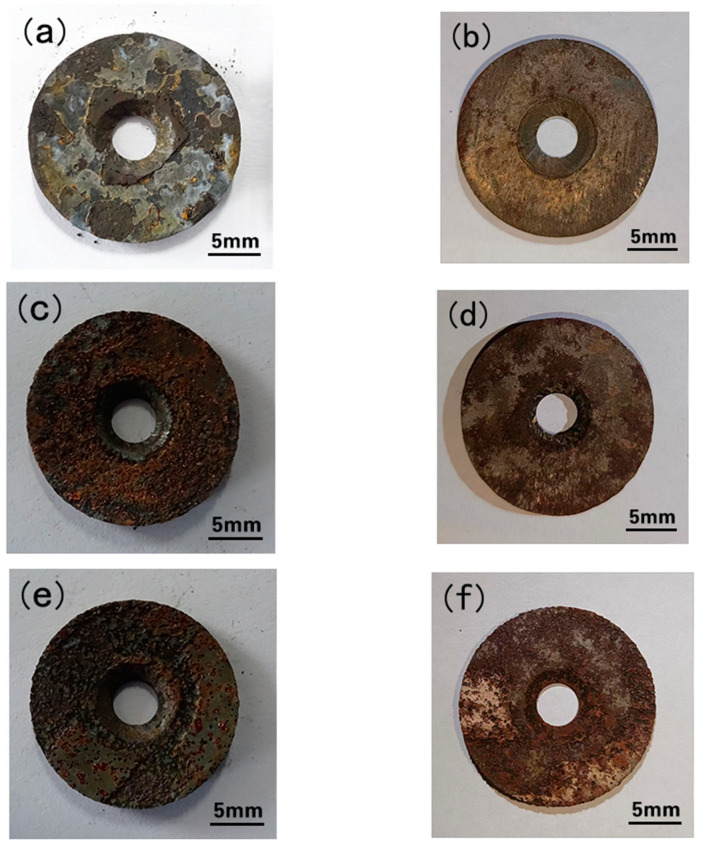
Macro morphologies of coupons with (**a**,**c**,**e**) and without (**b**,**d**,**f**) the corrosion film after flow loop tests at 20 °C (**a**,**b**), 50 °C (**c**,**d**), and 60 °C (**e**,**f**).

**Figure 4:**
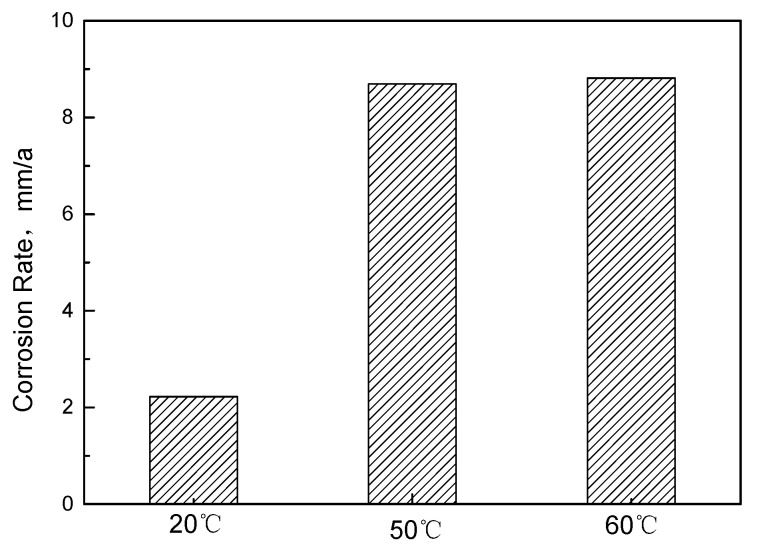
Corrosion rate of antibacterial alloy steel measured under different temperatures.

Figure 3 shows the macro morphology of couples with and without corrosion film after the 168 h flow loop tests. At 20 °C, the surface of antibacterial alloy steel is covered with relatively complete corrosion product film. The corrosion film of the antibacterial alloy steel showed obvious damage at 50 °C and 60 °C. There is obvious pitting corrosion on the surface of the sample, and the pitting corrosion is much more serious at 60 °C. It can be seen that the pits on the surface of antibacterial steel gradually increase when the temperature changes. Figure 4 shows the corrosion rate results at different temperatures. The corrosion rate of anti−bacterial alloy steel is lower at 20 °C. When the temperature rises to 50 °C and 60 °C, the corrosion rate increases rapidly. The above results suggest that temperature promotes the corrosion of antibacterial alloy steel.

**Figure 5:**
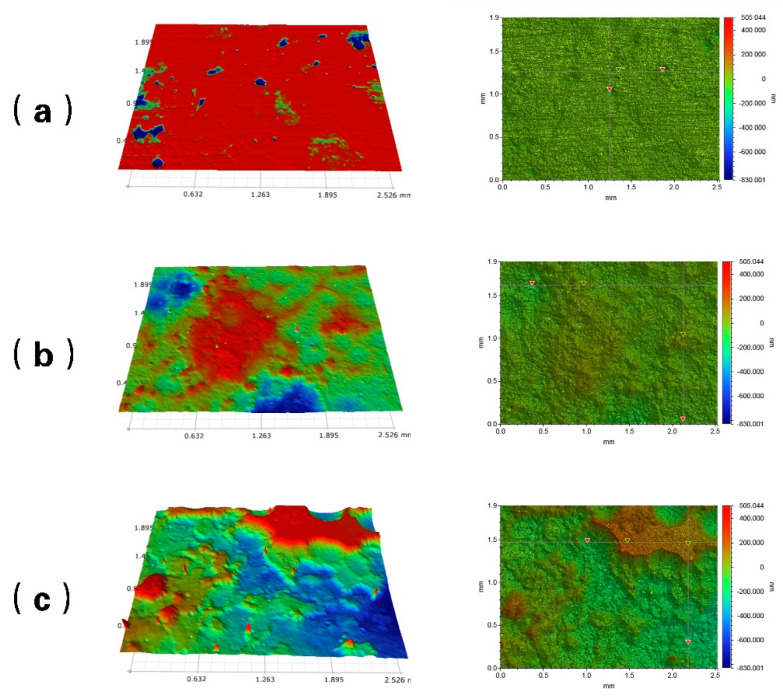
Macro topographies of the coupons after flow loop tests at 20 °C (**a**), 50 °C (**b**), and 60 °C (**c**).

Figure 5 shows the macro topographies of the coupons after the 168 h flow loop tests. At 20 °C, there are no visible holes or pits on the sample surface, thus predominately exhibiting uniform corrosion. At 50 °C, the sample surface is rough with some shallow pitting. There is serious localized corrosion at 60 °C, and the corrosion pits are deep. It can be seen that the pitting risk of antibacterial steel increases significantly with increasing temperature. By comparing the macroscopic morphology of the corrosion film in Figure 4, it can be seen that with the increase of temperature, the densification of the corrosion product film on the sample surface deteriorates. Seo et al. found that when the corrosion solution contains chloride ions, the copper ions in the substrate react with Cl^−^ in polarization, forming porous corrosion films [23]. When the temperature increases, the ionic activity in the solution increases, and the corrosive medium easily enters the substrate through the damaged point of the corrosion film, accelerating the formation of pitting corrosion. On the other hand, Cu has a higher electrode potential, which may aggravate the surface electrochemical reaction of the substrate in an acidic gas environment [24]. Thus, it is found that the corrosion resistance of antibacterial steel in CO_2_ environments is related to temperature.

**Figure 6:**
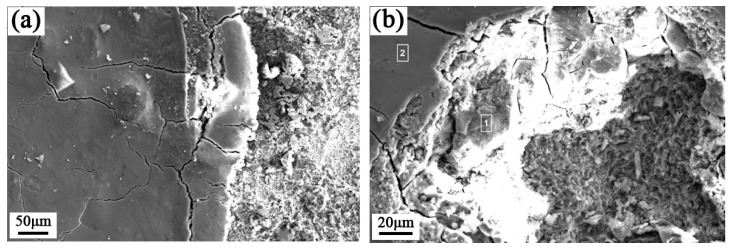

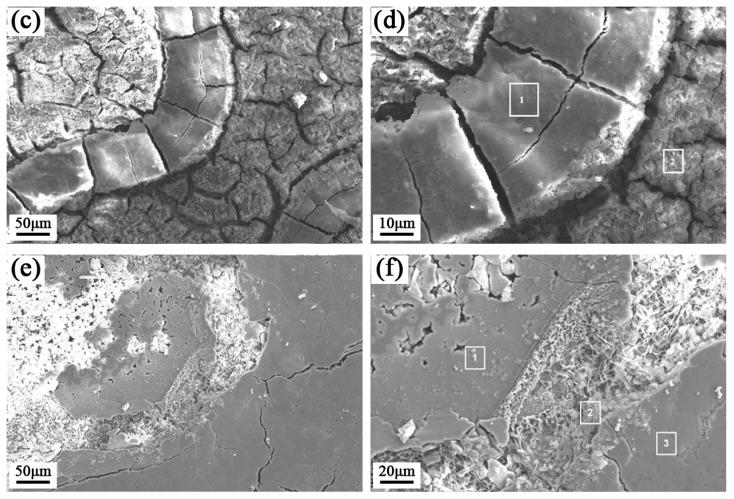
The microscopic morphology of the corrosion film after the 168 h flow loop tests at 20 °C (**a**,**b**), 50 °C (**c**,**d**), and 60 °C (**e**,**f**).

The microstructure of the corrosion film after multiphase flow corrosion is shown in Figure 6. At 20 °C, a dense corrosion film was formed on the sample surface due to decrease of ion activity at lower temperature. The numbers of Figure 6b,d,f were EDS test areas. According to EDS analysis results (as shown in Table 4), it can be inferred that the film with better protective effect is iron oxide or FeCO_3_. A layer of corrosion products still appeared under multiphase flow corrosion conditions of 50 °C and 60 °C, where the corrosion product film had partially fallen off, and a variety of local corrosion products piled up in disorder, thus showing the coexistence of various corrosion forms. EDS results show that the corrosion products are mainly composed of Fe, Cr, and O, forming through pores and becoming fast channels for corrosive media and ions. As temperature increases, the activity of iron ion increases [25,26], leading to a formation of porous corrosion film on the surface, and the protection of the corrosion film decreases under the action of fluid flow. Therefore, this is also the main reason for the rapid increase of corrosion rate at high temperature.

**Figure 7:**
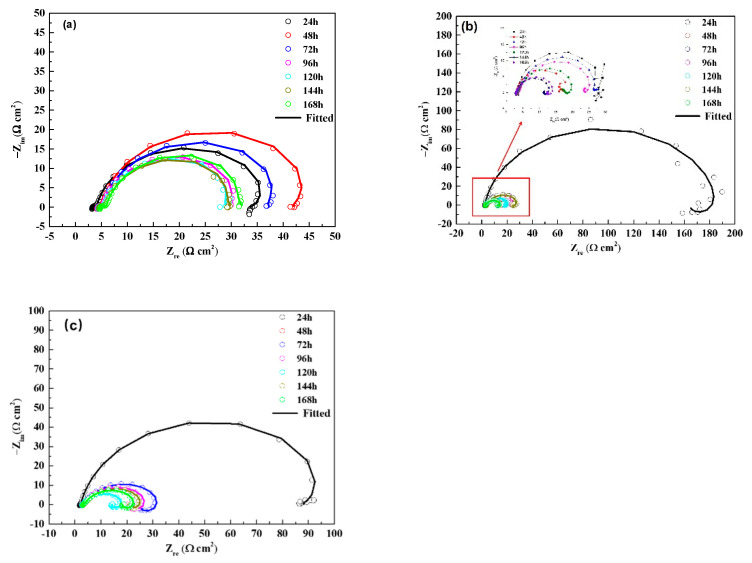
Nyquist plots of EIS results after the 168 h flow loop tests at (**a**) 20 °C, (**b**) 50 °C, and (**c**) 60 °C.

**Figure 8:**
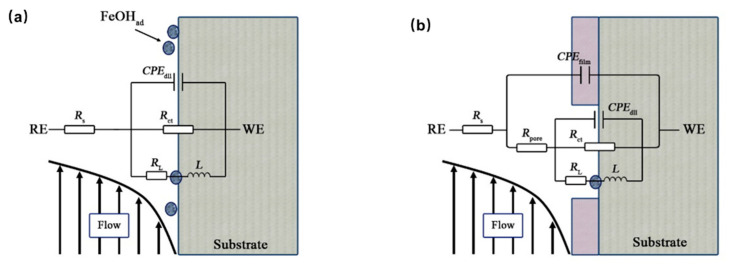
Equivalent circuits used for modeling the EIS results in Figure 8. (**a**) No film, (**b**) With film.

Electrochemical impedance spectroscopy (EIS), a type of electrochemical measurement method, can quickly obtain the information regarding the metal/solution interface. It is often used to study the evolution mechanism and corrosion development process of corrosion product films. In order to clarify the corrosion mechanism of samples in CO_2_ environment of different temperatures, EIS was used to conduct realtime monitoring during the corrosion process. As shown in Figure 7, the inductive reactance arcs in the low frequency became more obvious with the increase of temperature. The increase of inductive reactance arc may be related to the adsorption and desorption process of corrosion intermediates in the interface region [27], indicating that the surface corrosion reaction is more intense at higher temperature. At 20 °C, the impedance arc of the sample increases first and then decreases with time, and tends to be stable after 96 h, which may be associated to the evolution of the corrosion product film. Figure 8 illustrates equivalent circuits of the EIS fitting. R_s_, CPE_film_, and R_pore_ denote the electrolyte solution resistance, constant phase element (CPE) used to fit the corrosion film capacitance, and resistance of the pores in the corrosion film, respectively. The electrochemical processes (see Table 5) at the interface are denoted by Rct and CPE_dl_, which represent the charge transfer resistance and the electric double−layer capacitance, respectively. RL and L were the inductive resistance and inductance, respectively. At the initial stage of corrosion, with the progress of corrosion, a protective corrosion product film is gradually formed on the surface of the sample, and the impedance increases accordingly. When the corrosion product film is deposited to a certain thickness, part of the corrosion product film may fall off due to the erosion caused by the fluid and the vulnerability of the corrosion product film itself, and the impedance arc may actually decrease. With the growth, dissolution, and spalling of the corrosion product film, the impedance value tends to be stable. The evolution process of the impedance spectrum is similar at 50 °C and 60 °C, but the difference is that at higher temperature, the corrosion reaction rate is higher, as is the film formation rate of corrosion products, so the impedance arc at 24 h is obviously larger than that at 20 °C. By comparing the impedance arcs at 168 h at three different temperatures, it is found that the higher the temperature is, the smaller the impedance arcs are, indicating the corrosion rate is higher at higher temperatures, which is consistent with the result of the hanging chip immersion. The main reasons are as follows: the effect of temperature on the corrosion rate of low alloy steel is mainly reflected in two aspects. On the one hand, with the increase of temperature, the chemical reaction rate increases, which will promote corrosion; on the other hand, with the rise of temperature, it is more conducive to the formation of dense and protective corrosion product film, which will inhibit corrosion. In the temperature range of 50–60 °C, the temperature plays a dominant role in promoting the corrosion rate, resulting in a higher corrosion rate at this temperature.

To sum up, the main anodic reactions of antibacterial steel in the multiphase flow corrosion environment are as follows:

(1) $$\mathrm{Fe}\to {\mathrm{Fe}}^{2+}+2e$$

(2) $$\mathrm{Cr}\to {\mathrm{Cr}}^{3+}+3e$$

(3) $$\mathrm{Cu}+{\mathrm{Cl}}^{-}\to \mathrm{CuCl}+e$$

Cathodic process in CO_2_–O_2_ coexisting environments include acid depolarization corrosion of CO_2_, and oxygen depolarization corrosion due to the presence of a small amount of O_2_:

(4) $$2H^{+}+2e$$

(5) $$O_{2}+4H^{+}+4e\to 2H_{2}O$$

Under the condition of CO_2_–O_2_, Fe in the antibacterial steel can directly react with OH to form Fe(OH)_3_ and Fe(OH)_2_, after forming a porous structure on the surface. The structure of Fe_2_O_3_ is complex and diverse, and the adhesion is very poor, making it difficult to form a dense corrosion product film. The corrosion ions in the solution easily penetrate into the pores, as the corrosion product film is loose and porous [28]. In the multiphase flow environment, the temperature and the presence of a small amount of O_2_ are the reasons for the intensification of the antibacterial corrosion [29].

(6) $${\mathrm{Fe}}^{3+}+3{\mathrm{OH}}^{-}=\mathrm{Fe}{\left(\mathrm{OH}\right)}_{3}$$

(7) $${\mathrm{Fe}}^{2+}+2{\mathrm{OH}}^{-}=\mathrm{Fe}{\left(\mathrm{OH}\right)}_{2}\;$$

(8) $$2\mathrm{FeO}\left(\mathrm{OH}\right)={\mathrm{Fe}}_{2}O_{3}+H_{2}O$$

(9) $${\mathrm{Fe}}^{2+}+{\mathrm{HCO}}_{3}^{-}={\mathrm{FeCO}}_{3}+H^{+}$$

(10) $${\mathrm{Cr}}^{3+}+3{\mathrm{OH}}^{-}=\mathrm{Cr}{\left(\mathrm{OH}\right)}_{3}$$

## 5. Conclusions

A large−scale multiphase flow loop, a homemade plane corrosion coupon, and a three−electrode probe were employed to conduct in situ corrosion and electrochemical measurements on an antibacterial alloy steel. At 20 °C, the corrosion product film on the antibacterial steel surface provides good protection. When the temperature reaches 50 °C and 60 °C, the pitting susceptibility of antibacterial steel increases sharply. This shows that in a CO_2_ multiphase flow environment, the application of antibacterial steel should consider not only the effect of temperature change on the corrosion of antibacterial steel, but also the pitting corrosion sensitivity of antibacterial steel caused by temperature change.

## Figures and Tables

**Table 1:** Chemical composition of antibacterial alloy steel.

C	Cr	Mo	Si	Mn	Cu	V	Ni	Al	Ti	Fe
0.057	1.970	0.170	0.160	0.290	0.560	0.072	0.59	0.021	0.022	Bal.

**Table 2:** Composition of the flow loop test solution (mg/L).

NaHCO_3_	Na_2_SO_4_	MgCl_2_	NaCl	CaCl_2_	SrCl_2_
32.98	92.38	427.77	17,065.67	19,037.2	2476.68

**Table 3:** The parameters of the flow loop tests.

No.	T (°C)	P_CO2_ (MPa)	O_2_ (ppb)	Velocity (ms^−1^)	t (h)
1	20	5	155	0.75	168
2	50	5	155	0.75	168
3	60	5	155	0.75	168

**Table 4:** EDS results of the corrosion scales after 168 h flow loop test.

T/°C	Area	O	Cr	Fe	Ca	Cu	Cl
20	1	15.20	1.90	82.90	−	−	−
	2	21.34	−	78.66	−	−	−
50	1	16.69	0.85	81.48	0.99	−	−
	2	28.53	31.10	15.05	10.13	15.19	−
60	1	18.75	0.05	73.96	−	1.56	5.68
	2	23.85	0.02	75.01	−	−	1.11

**Table 5:** Values of the elements of the equivalent circuit in Figure 8 to fit the impedance spectra of Figure 7.

Conditions	R_s_ (Ω·cm^2^)	CPE_film_ (Ω^−1^∙s^n^∙cm^−2^)	CPE_film_−n	R_pore_ (Ω·cm^2^)	CPE_dl_ (Ω^−1^∙s^n^∙cm^−2^)	CPE_dl_ n	R_ct_ (Ω·cm^2^)	R_L_ (Ω⋅cm^2^)	L (H⋅cm^−2^)
20 °C−24 h	3.32	4.76 × 10^−4^	0.87	1.08	2.19 × 10^−3^	0.91	35.67	163.4	70.16
20 °C−48 h	4.28	9.56 × 10^−4^	0.81	1.03	4.90 × 10^−3^	0.93	44.84	203.1	190.3
20 °C−72 h	4.45	1.22 × 10^−3^	0.79	1.04	6.71 × 10^−3^	0.92	39.78	159.2	126.5
20 °C−96 h	4.32	1.67 × 10^−3^	0.76	1.04	8.68 × 10^−3^	0.92	31.28	110.5	81.04
20 °C−120 h	4.47	1.74 × 10^−3^	0.74	1.15	1.05 × 10^−2^	0.92	30.49	101.6	76.57
20 °C−144 h	4.60	1.67 × 10^−3^	0.72	1.22	1.29 × 10^−2^	0.89	32.91	90.28	56.64
20 °C−168 h	4.87	1.98 × 10^−3^	0.72	1.16	1.41 × 10^−2^	0.88	37.87	84.51	57.23
50 °C−24 h	2.53	−	−	−	4.87 × 10^−4^	0.91	185.4	1271	4350
50 °C−48 h	3.46	−	−	−	5.87 × 10^−3^	0.75	16.82	47.72	8.93
50 °C−72 h	3.28	−	−	−	4.64 × 10^−3^	0.80	34.14	80.87	13.84
50 °C−96 h	3.71	5.24 × 10^−3^	0.81	2.10 × 10^−3^	3.44 × 10^−2^	0.80	31.23	58.28	25.79
50 °C−120 h	3.33	4.75 × 10^−4^	1.00	0.37	5.15 × 10^−3^	0.81	19.26	52.86	23.88
50 °C−144 h	3.71	4.27 × 10^−4^	1.00	0.43	4.15 × 10^−3^	0.79	28.3	78.45	37.4
50 °C−168 h	2.98	−	−	−	4.68 × 10^−3^	0.80	13.68	30.99	6.54
60 °C−24 h	1.88	7.03 × 10^−5^	1.00	0.96	4.18 × 10^−4^	0.86	103	502.6	96.61
60 °C−72 h	3.15	−	−	−	1.72 × 10^−3^	0.74	32.29	63.07	29.82
60 °C−96 h	3.11	−	−	−	1.97 × 10^−3^	0.72	28.12	53.89	14.66
60 °C−120 h	2.82	2.30 × 10^−3^	0.70	10.16	9.79 × 10^−4^	0.36	8.32	1.63	0.9
60 °C−144 h	3.29	4.43 × 10^−5^	1.00	0.71	1.94 × 10^−3^	0.73	24.45	59.15	16.37
60 °C−168 h	3.14	4.96 × 10^−5^	1.00	0.78	1.75 × 10^−3^	0.75	21.34	44.99	10.83

## Data Availability

Not applicable.
